# The Involvement of Acetaldehyde in Ethanol-Induced Cell Cycle Impairment

**DOI:** 10.3390/biom6020017

**Published:** 2016-03-31

**Authors:** Marc A. Scheer, Katrina J. Schneider, Rochelle L. Finnigan, Eamon P. Maloney, Mark A. Wells, Dahn L. Clemens

**Affiliations:** 1 Department of Internal Medicine, University of Nebraska Medical Center, Omaha, NE 68105, USA; Scheer77@aol.com (M.A.S.); km0308@hotmail.com (K.J.S.); rochelle.finnigan@yahoo.com (R.L.F.); epmaloney@gmail.com (E.P.M.); wellsma@gmail.com (M.A.W.); 2 Nebraska and Western Iowa Veterans Administration Medical Center, University of Nebraska Medical Center, Omaha, NE 68105, USA; 3 Fred and Pamela Buffet Cancer Center, University of Nebraska Medical Center, Omaha, NE 68105, USA

**Keywords:** acetaldehyde, cyclin-dependent kinases, cyclin-dependent kinase inhibitors, cell cycle arrest, ethanol metabolism

## Abstract

Background: Hepatocytes metabolize the vast majority of ingested ethanol. This metabolic activity results in hepatic toxicity and impairs the ability of hepatocytes to replicate. Previous work by our group has shown that ethanol metabolism results in a G2/M cell cycle arrest. The intent of these studies was to discern the roles of acetaldehyde and reactive oxygen, two of the major by-products of ethanol metabolism, in the G2/M cell cycle arrest. Methods: To investigate the role of ethanol metabolites in the cell cycle arrest, VA-13 and VL-17A cells were used. These are recombinant Hep G2 cells that express alcohol dehydrogenase or alcohol dehydrogenase and cytochrome P450 2E1, respectively. Cells were cultured with or without ethanol, lacking or containing the antioxidants *N*-acetylcysteine (NAC) or trolox, for three days. Cellular accumulation was monitored by the DNA content of the cultures. The accumulation of the cyclin-dependent kinase, Cdc2 in the inactive phosphorylated form (p-Cdc2) and the cyclin-dependent kinase inhibitor p21 were determined by immunoblot analysis. Results: Cultures maintained in the presence of ethanol demonstrated a G2/M cell cycle arrest that was associated with a reduction in DNA content and increased levels of p-Cdc2 and p21, compared with cells cultured in its absence. Inclusion of antioxidants in the ethanol containing media was unable to rescue the cells from the cell cycle arrest or these ethanol metabolism-mediated effects. Additionally, culturing the cells in the presence of acetaldehyde alone resulted in increased levels of p-Cdc2 and p21. Conclusions: Acetaldehyde produced during ethanol oxidation has a major role in the ethanol metabolism-mediated G2/M cell cycle arrest, and the concurrent accumulation of p21 and p-Cdc2. Although reactive oxygen species are thought to have a significant role in ethanol-induced hepatocellular damage, they may have a less important role in the inability of hepatocytes to replace dead or damaged cells.

## 1. Introduction

The liver is the primary site of ethanol metabolism. The by-products of this metabolism can result in hepatotoxicity. Additionally, a number of studies have demonstrated that ethanol metabolism delays or impairs the replication of hepatocytes and cultured hepatic cells [1,2,3,4]. Because of this, chronic ethanol consumption not only causes cell damage and death, but it also impairs the ability of the liver to replace damaged cells and respond appropriately to injury.

There are two pathways by which damaged or dead hepatocytes, can be replaced [5,6]. Replication of existing mature hepatocytes normally replaces damaged or dead hepatocytes [7]. In many chronic liver diseases, the replication of mature hepatocytes is inhibited. In these instances, replacement of dead or damaged hepatocytes occurs by a population of bipotential hepatic cells known as hepatic progenitor cells or oval cells [8,9,10,11]. Because hepatic progenitor cells are bipotential and can differentiate into either bile ductular epithelium or hepatocytes, it has been proposed that activation of hepatic progenitor cells not only results in the replacement of hepatocytes, but proliferation of ductals is also increased [9,12,13]. In patients suffering from alcoholic liver disease, it has been demonstrated that portal inflammation, fibrosis, and cirrhosis correlate with the increased expression of ductular epithela [12]. Thus, increased expression of ductular epithela may precede the accumulation of fibrotic tissue in the liver. Therefore, impairment of normal hepatocyte replication by ethanol metabolism and activation of hepatic progenitor cells may have a role in the fibrotic scarring characteristics of alcoholic liver disease.

In previous studies, we have shown that ethanol metabolism by recombinant Hep G2 cells, engineered to efficiently metabolize ethanol, results in impaired cellular replication [1,2,14]. This impairment in cellular replication is, at least partially, the result of a G2/M cell cycle arrest. Furthermore, this G2/M cell cycle arrest is mediated, at least in part, by the activation of cell cycle check point kinases and the accumulation of the cyclin dependent kinase, Cdc2, in the inactive, phosphorylated form [1,15].

The activity of cyclin-dependent kinases is tightly regulated throughout the cell cycle to ensure smooth progression of cellular replication. Cdc2 activity is required for the transition from the G2 to M-phase of the cell cycle [16]. Cdc2 activity is regulated both positively and negatively by phosphorylation [17]. Throughout the majority of the cell cycle, Cdc2 is maintained in an inactive state. This is accomplished by phosphorylation at threonine 14 (Thr 14) and tyrosine 15 (Tyr 15). Phosphorylation of these amino acids hinders the binding of ATP to the kinase; thus, rendering Cdc2 inactive [18].

Using the recombinant Hep G2 cell lines, VA-13 that selectively metabolize ethanol via the alcohol dehydrogenase (ADH) pathway and VL-17A cells that express both alcohol dehydrogenase and cytochrome P450 2E1 (CPY2E1), we have investigated the involvement of acetaldehyde and reactive oxygen species in the ethanol metabolism-mediated cell cycle arrest. The results of these studies demonstrated that antioxidants were unable to protect either of these cell lines from the ethanol metabolism-mediated impairment of cellular replication. This impairment is characterized by accumulation of cells at the G2/M transition of the cell cycle, increased expression of the cyclin dependent kinase inhibitor p21, and increased accumulation of Cdc2 in the inactive phosphorylated form (p-Cdc2). Lastly, we showed that direct treatment of these cells with acetaldehyde results in increased accumulation of p-Cdc2 and p21. Collectively, these results indicate that acetaldehyde is the primary mediator of this cell cycle arrest and impairment in cellular replication.

## 2. Results

We have previously shown that ethanol metabolism impairs the replication of recombinant hepatic cells by arresting the cells at the G2/M transition of the cell cycle. This cell cycle arrest is mediated, at least in part, by an increase in the level of the inactive phosphorylated form of the cyclin-dependent kinase, Cdc2 [1]. To delineate the specific by-products of ethanol metabolism responsible for the cell cycle arrest and impaired cellular replication, we used VA-13 cells that express ADH, VL-17A cells that express both ADH and cytochrome CYP2E1, and control VI-7 cells, which are stably transfected with the empty vector and do not efficiently express ADH or CYP2E1 [2].

To determine the contribution of the various by-products of ethanol metabolism in the ethanol metabolism-mediated impairment of cellular replication, we first compared the growth of VA-13 and VL-17A cells. Cells were cultured in the absence or presence of 25 mM ethanol for three days and cell accumulation was determined by the DNA content of the cultures. There was no significant difference in the reduction in cell accumulation between these two cell lines. The accumulation of VA-13 and VL-17A cells was reduced by 37.1% ± 3.7% and 41.5% ± 2.9%, respectively. As expected, ethanol had no effect on the accumulation of the VI-7 cells, which do not efficiently metabolize ethanol.

To determine the by-product(s) of ethanol metabolism responsible for this inhibition of cell accumulation in the cultures, we next investigated the role of reactive oxygen species (ROS). To accomplish this, cells were cultured in control media, or media containing ethanol with or without the antioxidant trolox, a synthetic form of Vitamin E. The results of these studies, shown in Table 1, revealed that the addition of trolox was unable to ameliorate the ethanol metabolism-mediated impairment in cell accumulation. Because different antioxidants have different modes of action, we repeated the experiment using the glutathione precursor NAC. Similarly, inclusion of NAC was unable to rescue cells from the ethanol-mediated impairment in cell accumulation.

**Figure 1:**
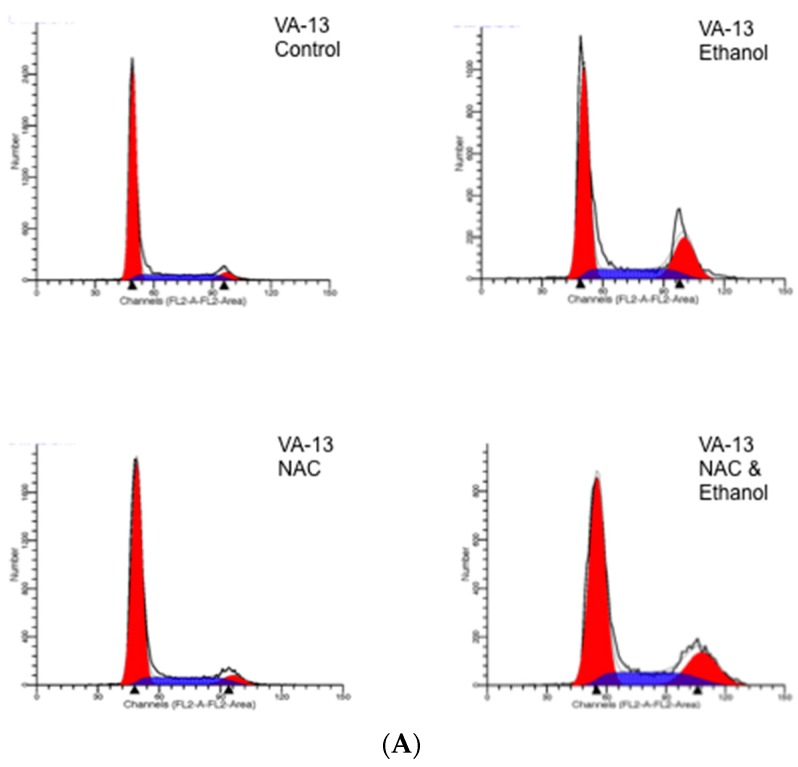

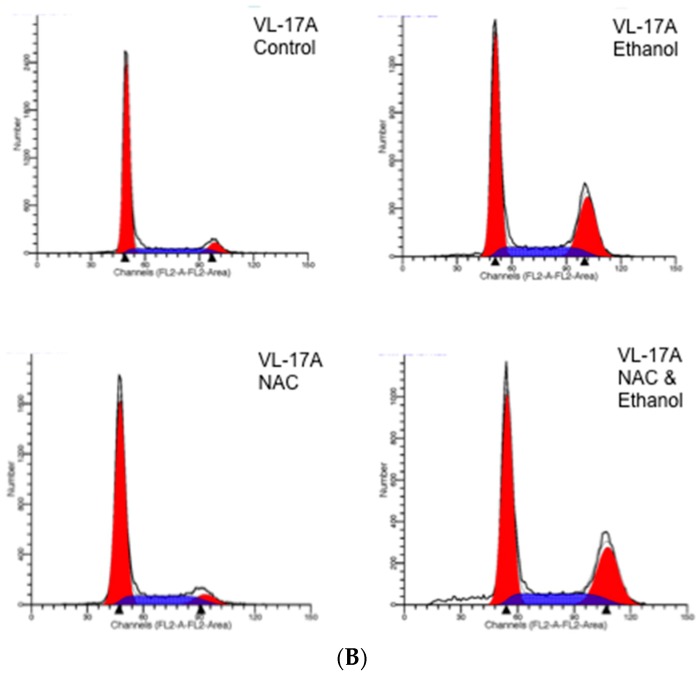
FACS analysis of cells cultured in the presence or absence of ethanol and NAC for three days. (**A**) VA-13 and (**B**) VL-17A cells were cultured in the presence or absence of media containing 25 mM ethanol and 1 mM NAC for three days. The cells were harvested and stained with Vindelov’s solution. DNA content, an indication of the stage of the cell cycle of the cells was determined by flow cytometry and analyzed using Modfit Software.

Because we had previously demonstrated that ethanol metabolism by recombinant Hep G2 cells resulted in a cell cycle arrest at the G2/M transition of the cell cycle, we next investigated if antioxidant treatment had an effect on cell cycle progression. Cells were cultured as described above and the cell cycle progression was monitored by flow cytometry. The results presented in Figure 1 revealed that the inclusion of the antioxidant NAC was not able to rescue either the VA-13 or VL-17A cells from this cell cycle arrest. The percentages of cells arrested at the G2/M transition in each treatment are presented in Table 2. Treatment with trolox yielded similar results in both cell lines.

**Figure 2:**
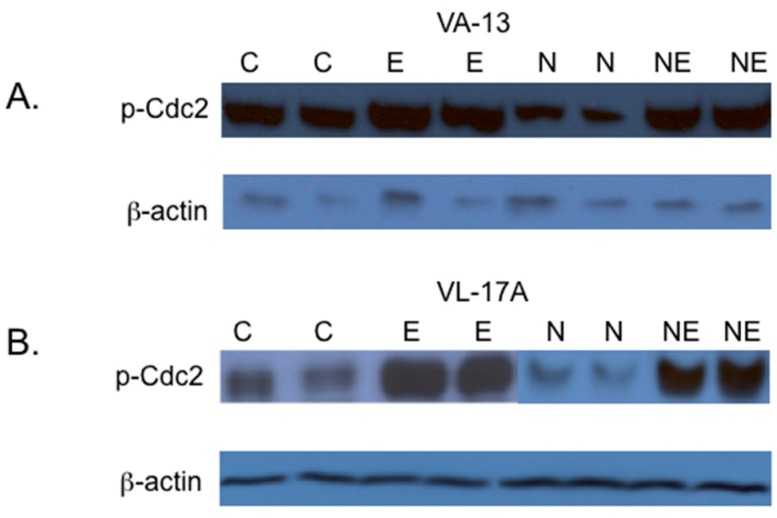
Effects of NAC on the phosphorylation of the cyclin-dependent kinase Cdc2. (**A**) VA-13 and (**B**) VL-17A cells were cultured in the presence or absence of 25 mM ethanol containing or lacking 1 mM NAC for three days. Lysates were prepared and immunoblot analysis performed. The results of these studies indicated that the inclusion of the antioxidant NAC in the growth media had no effect on the ethanol metabolism-mediated accumulation of Cdc2 in the inactive, phosphorylated form. (C = control cells, E = cells cultured in the presence of ethanol, N = cells cultured in the presence of NAC, and NE = cells cultured in the presence of NAC and ethanol).

**Figure 3:**
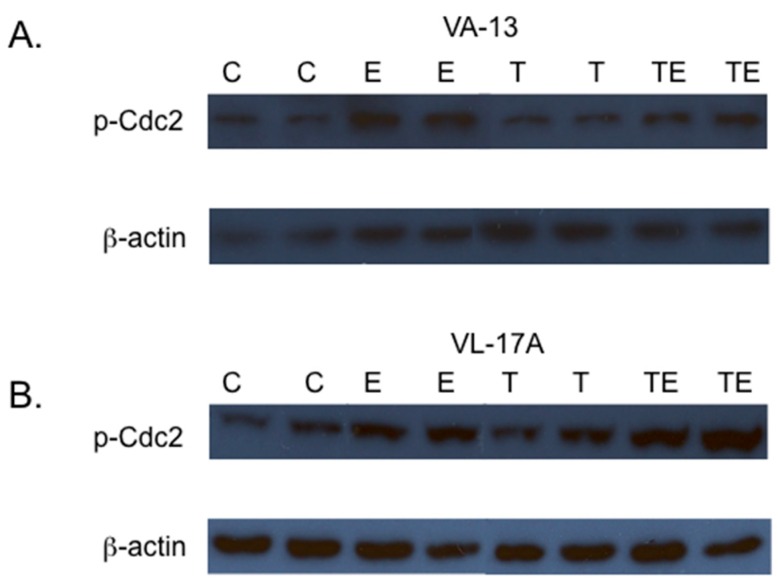
Effects of trolox on the phosphorylation of the cyclin-dependent kinase Cdc2. (**A**) VA-13 and (**B**) VL-17A cells were cultured in the presence or absence of 25 mM ethanol containing or lacking 50 µM trolox for three days. Lysates were prepared and immunoblot analysis performed. The results of these studies indicated that the inclusion of the antioxidant trolox in the growth media had no effect on the ethanol metabolism-mediated accumulation of Cdc2 in the inactive, phosphorylated form. (C = control cells, E = cells cultured in the presence of ethanol, T = cells cultured in the presence of trolox, and TE = cells cultured in the presence of trolox and ethanol).

In eukaryotic cells, activation of the cyclin dependent-kinase, Cdc2 is required for the transition from the G2 to M phase of the cell cycle. Cdc2 is inactivated by phosphorylation at Tyr 15. Because the VA-13 and VL-17A cells were arrested at the G2/M transition, we next investigated if increased levels of Cdc2 phosphorylated at Tyr 15 (p-Cdc2) were associated with this cell cycle arrest. The results of these studies showed that inclusion of ethanol to the growth media resulted in an increase in p-Cdc2 in both VA-13 cells (Figure 2 and Figure 3) and VL-17A cells (Figure 2 and Figure 3). Additionally, our data indicated that the increase in p-Cdc2 was also present in VA-13 cells and VL-17A cells cultured in ethanol and either NAC (Figure 2) or trolox (Figure 3). Because treatment with the antioxidants did not ameliorate the impaired cell accumulation, the cell cycle arrest, or the increase in p-Cdc2, it appeared that reactive oxygen species were not responsible for the ethanol metabolism-mediated impairment in cellular replication.

**Figure 4:**
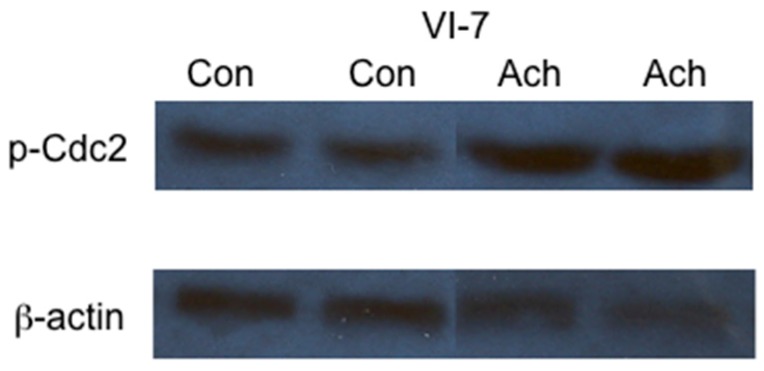
Effects of acetaldehyde on the phosphorylation of the cyclin-dependent kinase Cdc2. VI-7 cells were cultured in the presence of 200 µM acetaldehyde and 0.75 mM cyanamide for 24 h. Lysates were prepared and immunoblot analysis performed. The results of these studies indicated that the inclusion of the acetaldehyde in the growth media resulted in increased levels of Cdc2 in the inactive, phosphorylated form. (Con = control cells, Ach = cells cultured in the presence of acetaldehyde).

Acetaldehyde, a toxic by-product of ethanol metabolism, has been implicated in a number of ethanol-associated dysfunctions. To investigate if acetaldehyde was involved in the ethanol metabolism-mediated impairment in cellular replication, we investigated the effects of acetaldehyde on Cdc2 phosphorylation. VI-7 cells were cultured in the presence of 200 µM acetaldehyde and 0.075 mM cyanamide for 24 h. Immunoblot analysis of lysates prepared from these cultures was performed to determine the level of p-Cdc2. The results, presented in Figure 4, demonstrated that acetaldehyde is sufficient for the accumulation of the inactive, phosphorylated form of Cdc2.

**Figure 5:**
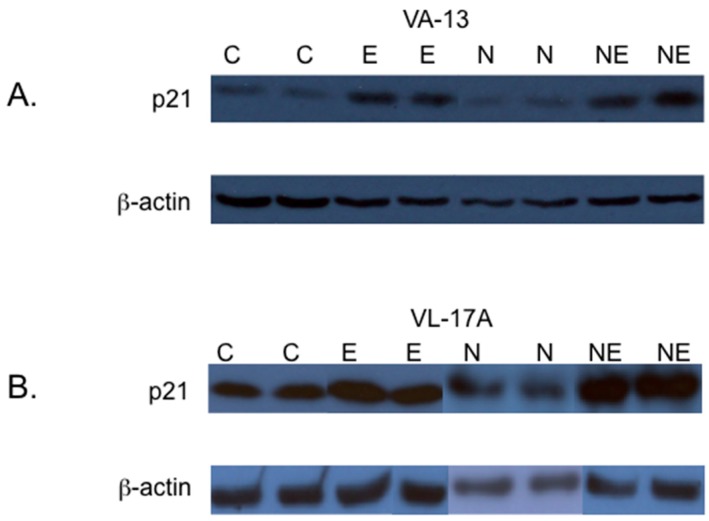
Effects of NAC on the expression of the cyclin dependent kinase inhibitor p21. (**A**) VA-13 and (**B**) VL-17A cells were cultured in the presence or absence of 25 mM ethanol containing or lacking 1 mM NAC for three days. Lysates were prepared and immunoblot analysis performed. The results of these studies indicated that the inclusion of the antioxidant NAC in the growth media had no effect on the ethanol metabolism-mediated increase in the expression of p21. (C = control cells, E = cells cultured in the presence of ethanol, N = cells cultured in the presence of NAC, and NE = cells cultured in the presence of NAC and ethanol).

**Figure 6:**
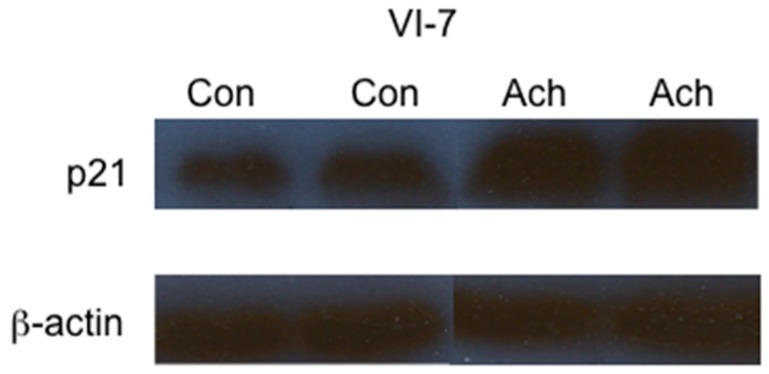
Effects of acetaldehyde on the expression of the cyclin-dependent kinase inhibitor p21. VI-7 cells were cultured in the presence of 200 µM acetaldehyde and 0.075 mM cyanamide for 24 h. Lysates were prepared and immunoblot analysis performed. The results of these studies indicated that the inclusion of acetaldehyde in the growth media resulted in increased expression of p21. (Con = control cells, Ach = cells cultured in the presence of acetaldehyde).

Although the accumulation of p-Cdc2 in cells metabolizing ethanol is likely a major contributor to the G2/M cell cycle arrest observed in VA-13 and VL-17A cells, we investigated if the cyclin-dependent kinase inhibitor p21 was also affected. The results, presented in Figure 5, demonstrate that ethanol metabolism results in increased levels of p21. Interestingly, inclusion of NAC in the growth media did not reduce this increase. To determine if acetaldehyde itself was sufficient to increase the levels of p21, we cultured VI-7 cells in the presence of acetaldehyde, as described above, and performed immunoblots (Figure 6). These results indicate that acetaldehyde is sufficient to cause increased expression of the cyclin-dependent kinase inhibitor p21. These findings further support the important role of acetaldehyde in the ethanol metabolism-mediated cell cycle impairment.

## 3. Discussion

The two major pathways of ethanol metabolism in the liver are catalyzed by the cytosolic enzyme ADH and the microsomal enzyme CYP2E1. Metabolism of ethanol by these pathways primarily results in the production of acetaldehyde and reactive oxygen species. Metabolism of ethanol by ADH is generally associated with the production of acetaldehyde, whereas ethanol metabolism by CPY2E1 is associated with the generation of reactive oxygen species. Both of these by-products have been implicated in a number of cellular dysfunctions [19,20,21,22,23,24,25].

Treatment of experimental animals with ethanol has been shown to impair the regeneration of injured tissues including the liver and pancreas [3,4,5,26,27,28,29]. Likewise, it has been shown that ethanol metabolism impairs the replication of hepatocytes and cultured hepatic cells [1,2,3,4]. Thus, when considering how ethanol metabolism damages an organ, it is necessary to consider both the toxic effects of ethanol metabolism, and the effects of ethanol metabolism on the repair and regeneration of the tissue.

Because ethanol metabolism can inhibit the repair of damaged tissue, we set out to determine the contribution of these two metabolic pathways and their resulting metabolic by-products to the impairment of cellular replication. To investigate the contribution of reactive oxygen species and acetaldehyde, we used two recombinant Hep G2 cell lines. The cell lines were VA-13, cells that selectively express ADH, and VL-17A, cells that express both ADH and CYP2E1. We reasoned that if reactive oxygen species were primarily responsible for this dysfunction, the ethanol metabolism-mediated impairment in cellular replication would be greater in VL-17A cells. Conversely, we reasoned that if acetaldehyde was responsible for this dysfunction, the impairment in cellular replication would be similar in both VA-13 and VL-17A cells.

To test this, we initially cultured VL-17A and VA-13 cells in the presence of 25 mM ethanol, (approximately 0.15% or twice the legal blood alcohol level in most states). We found that there was no difference in the effects of ethanol metabolism between the two cell lines, the accumulation of cells being inhibited to a similar degree in both cell lines. To determine the contribution of reactive oxygen species, we included antioxidants in the growth media. Surprisingly, the glutathione precursor, NAC and the synthetic form of Vitamin E, trolox, were unable to rescue either the VA-13 or VL-17A cells from this impairment. These results indicated that reactive oxygen species were not the primary metabolic by-product responsible for the ethanol metabolism-mediated impairment in cellular accumulation.

Activity of the cyclin-dependent kinase Cdc2 is required by eukaryotic cells to pass from the G2 to M phases of the cell cycle. Phosphorylation of Cdc2 at Tyr 15 blocks ATP binding to the kinase rendering it inactive. We have previously shown that the ethanol metabolism-mediated impairment in the ability of these cells to replicate is associated with cell cycle arrest at the G2/M transition of the cell cycle and an increase in the level of p-Cdc2 [1]. To further test the role of acetaldehyde and reactive oxygen species in this impairment, we analyzed the cell cycle progression in cells cultured in media containing ethanol and cells cultured in media containing ethanol and antioxidants. Again, there was very little difference between the two cell lines. The inclusion of ethanol in the growth media resulted in a G2/M cell arrest, and antioxidants were unable to rescue the cells. Additional studies revealed that inclusion of ethanol in the growth media of these cells resulted in increased levels of Tyr 15 phosphorylated Cdc2. Again, the addition of antioxidants was unable to ameliorate this impairment.

To demonstrate if acetaldehyde was capable of mediating this cell cycle arrest, we cultured the recombinant Hep G2 cells, VI-7, in the presence of acetaldehyde. VI-7 cells do not possess ADH or CYP2E1 activity and are unable to metabolize ethanol. Treatment of these cells with acetaldehyde resulted in increased levels of p-Cdc2, indicating that acetaldehyde mediated this dysfunction.

Although accumulation of Cdc2 in the inactive state, at least in part, explains the observed ethanol metabolism-mediated cell cycle arrest, we investigated if other factors that inhibit cell cycle progression were affected by ethanol metabolism. We had observed that p53 was affected in VA-13 and VL-17A cells. The cyclin-dependent kinase inhibitor p21 is a direct target of p53 [30]. Increased expression of p21 in a number of systems is associated with cell cycle arrest. Because of this, we investigated if ethanol metabolism increased the expression of the cyclin-dependent kinase inhibitor p21. The results of these studies revealed that treatment with either ethanol or acetaldehyde increased the expression of p21.

p21 belongs to the Cip/Kip family of cyclin-dependent kinase inhibitors and is associated with cell cycle arrest and cellular senescence [31]. The expression of p21 not only inhibits the activity of cyclin-dependent kinases, it also indirectly inhibits the expression of a number of genes involved in mitosis, as well as DNA replication and repair [32]. Thus, the ethanol metabolism-mediated increase in the expression of p21 likely has a role in the observed cell cycle arrest.

After DNA damage, the cell cycle of many cells is arrested at the G2 phase of the cell cycle [33]. Acetaldehyde can damage DNA by covalently binding to DNA forming adducts [34]. Detection of these adducts by the cell activates the checkpoint kinases and arrests the cell cycle progression. In previous studies, we demonstrated that ethanol metabolism by VA-13 cells activates ataxia-telangiectasia mutated (ATM), which in turn activates the checkpoint kinase Chk2 [15]. Chk2 can phosphorylate Cdc25c the phosphatase that activates Cdc2. Phosphorylation of Cdc25c results in its sequestration in the cytoplasm where it is unable to activate Cdc2. This results in a cell cycle arrest at the G2/M transition. Checkpoint kinases can also activate p53, which, in turn, induces expression of p21. Increased expression of p21 can result in G2 cell cycle arrest. Furthermore, the maintenance of the cell cycle arrest requires p53 and p21 [33]. Thus, it is likely that acetaldehyde adducts DNA, activating the checkpoint kinases, which decreases Cdc2 activity and increases the p53-dependent expression of p21 ultimately resulting in cell cycle arrest.

Increased expression of p21 in the liver can have negative or positive repercussions. In human beings suffering from alcoholic hepatitis, p21 is upregulated in response to hepatic injury [35]. It has been demonstrated that increased expression of p21 in the liver of individuals suffering from either nonalcoholic fatty liver disease or alcoholic liver disease is associated with increased fibrosis and a poor outcome [36,37]. This may be because several genes induced by p21 encode secreted proteins with paracrine effects that induce cell growth and inhibit apoptosis [38]. Thus, although expression of p21 is associated with impaired cellular replication, expression of p21 may also confer a survival benefit on adjacent cells. These factors may be acting on hepatic stellate cells, the activation of which results in the increased synthesis and deposition of collagen.

Cell cycle arrest of cells in response to DNA damage is generally thought to be beneficial. Koteish *et al.* demonstrated that p21 is upregulated in the livers of ethanol-fed mice after partial hepatectomy when compared with pair-fed mice. The increased expression of p21 is associated with increased expression of the mitogen activated protein kinase p38 and the nuclear localization of phosphorylated STAT 3. The authors suggested that increased expression of these factors, which inhibit cell cycle progression, benefit the survival of cells by delaying the cell cycle progression until DNA lesions can be repaired [4]. Thus, increased expression of p21 and cell cycle arrest may in some cases be a protective mechanism.

In the liver, impairment of hepatocyte replication can lead to activation of bipotential hepatic precursor cells. Because these cells are bipotential, their activation results in the production of hepatocytes and biliary epithelium. The expansion of biliary epithelium may have a role in the initiation of fibrosis. In patients suffering from alcoholic liver disease, it has been shown that increased expression of ductular epithela correlates highly with portal inflammation, fibrosis, and cirrhosis [12]. Thus, the inability of hepatocytes to replicate in response to injury may have pathologic effects.

In summary, we have demonstrated that the ethanol metabolism-mediated G2/M cell cycle arrest observed in recombinant Hep G2 cells is primarily caused by acetaldehyde, the by-product of ethanol metabolism. The cell cycle arrest is characterized by the accumulation of the cyclin-dependent kinase Cdc2 in the inactive phosphorylated form, and increased expression of the cyclin dependent kinase inhibitor p21. The ethanol induced cell cycle arrest may be a physiologic response to DNA damage caused by the formation of acetaldehyde-DNA adducts. This cell cycle arrest provides time for repair of the damage before completion of DNA synthesis and mitosis.

## 4. Materials and Methods

### 4.1. Cell Culture

VA-13 cells are recombinant Hep G2 cells that have been stably transfected to express alcohol dehydrogenase (ADH), VL-17A cells are recombinant Hep G2 cells that have been stably transfected to express both alcohol dehydrogenase and cytochrome P450 2E1 (CYP2E1). VI-7 cells are recombinant Hep G2 cells transfected with the empty vector and do not metabolize ethanol. The construction and characterization of these cell lines has been previously described [2,14]. VA-13 and VI-7 cells were maintained in DMEM containing high glucose and supplemented with 10% fetal bovine serum, 2 mM glutamine, 400 µg/mL zeocin, 50 µg/mL gentamicin, 100 units/mL penicillin and 100 µg/mL streptomycin (complete DMEM). VL-17A cells were maintained in the above media that also contained 400 µg/mL G418. For ethanol and acetaldehyde experiments cells were cultured as above, but 25 mM HEPES [*N*-(2-hydroxyethyl) piperazine-*N*-(2-ethanesulfonic acid)] pH 7.2 was included in the growth media of all flasks. Additionally, 25 mM ethanol was included in the growth media of cells exposed to ethanol, and 200 µM acetaldehyde and 75 µM cyanamide (an aldehydedehydrogenase inhibitor) was included in the growth media of cells exposed to acetaldehyde. In studies in which antioxidants were used either 1 mM *N*-acetylcysteine (NAC) or 50 µM trolox was included in the culture media. During ethanol and acetaldehyde experiments, all flasks were tightly sealed to minimize evaporation of ethanol and acetaldehyde.

### 4.2. Immunoblotting

Cells were removed from the flasks by trypsinization and collected by centrifugation. The cell pellets were lysed in RIPA buffer (50 mM Tris pH 7.4, 1% NP-40, 0.25% Na deoxycholate, 150 mM NaCl, 1 mM EDTA, 1 mM phenylmethylsulfonyl fluoride, 1 mM NaVO_4_, and 1 mM NaF). Complete mini protease cocktail was added to the buffer, as recommended by the manufacturer (Roche, Indianapolis, IN, USA). The protein concentrations of the lysates were determined using the Bio-Rad protein reagent (Bio-Rad Laboratories, Hercules, CA, USA) with bovine serum albumin as a standard. Thirty micrograms of protein from each sample was separated by sodium dodecyl sulfate-polyacrylamide gel electrophoresis. Proteins were electroblotted onto polyvinyl difluoride (PVDF) membranes in 25 mM Tris, 192 mM glycine, and 20% methanol at 100 volts for 60 min at 4 °C. The membranes were blocked in TBST (20 mM Tris pH 7.6, 136 mM NaCl, and 0.1% Tween 20) containing 5% nonfat dry milk for 1 h at room temperature, and incubated at 4 °C overnight with the primary antibody diluted in the TBST/milk solution. The membranes were washed in TBST and incubated for 1 h with a peroxidase-conjugated secondary antibody diluted in TBST/milk, washed, and the proteins visualized by chemiluminescence using the SuperSignal West Pico chemiluminescent substrate (Pierce, Rockford, IL, USA). The resulting bands were quantified by densitometry using Quantity One software (Bio Rad). Antibodies used were anti-p21, anti-phospho-Cdc2 (Tyr15) (Upstate Biotechnology, Lake Placid, NY, USA), and anti-β-actin (Sigma, St. Louis, MO, USA).

### 4.3. DNA Determination and Cell Cycle Analysis

Cells were cultured as described above, collected after typsinization, and the quantity of DNA in each culture was determined using Hoechst 33258, as described by Labarca and Pagigen [39]. Briefly, after sonication, aliquots of each lysate were diluted to a final volume of 1 mL in PBS, and 1 µL of 0.1 g/mL of the Hoechst was added. DNA was quantitated by fluorescence (excitation, 356 nm; emission, 458 nm) and compared with a DNA standard.

Cell cycle state was determined by flow cytometry, after staining cells with Vindelov’s solution (TBS containing 0.001% RNAse A, 0.075% propidium iodide, and 0.001% NP-40), using a FACaliber flow cytometer and analyzed using Modfit Software (Verity Software House, Topshan, ME, USA) [1].

## 5. Conclusions

Ethanol metabolism by hepatocytes results in a number of biochemical changes and the production of toxic by-products. We have shown that one of the toxic by-products, acetaldehyde, also effects the ability of cells to replicate. This ethanol metabolism-mediated impairment in the ability of hepatocytes to replicate alters the regenerative capability of the liver. Thus, ethanol metabolism not only causes tissue damage in the liver, but also alters the ability of the liver to respond appropriately to the damage. Because of this, both the toxic effects of ethanol metabolism, as well as alterations in the ability of hepatocytes to regenerate must be considered when evaluating the effects of ethanol on the liver.

## Figures and Tables

**Table 1:** DNA content of cells cultured in the presence or absence of 25 mM ethanol and antioxidants.

Cells	Control	EtOH	EtOH and NAC	EtOH and Trolox
VL-17A	77.1 ± 4.8 ^a^	45.2 ± 3.4 ^a^	42.1 ± 4.9 ^a^	39.4 ± 4.1 ^a^
VA-13	78.3 ± 3.4 ^a^	49.3 ± 4.8 ^a^	48.1 ± 5.1 ^a^	41.9 ± 4.2 ^a^

^a^ Data is presented as the mean micrograms of DNA ± the SE. *n* = 4.

**Table 2:** Percentage of cells in the G2/M phase of the cell cycle after growth for three days in the presence or absence of 25 mM ethanol.

Cells	Control	EtOH	NAC	NAC and EtOH
VA-13	6.8 ± 0.5 ^a^	21.8 ± 0.15 ^a^	6.8 ± 0.3 ^a^	19.6 ± 0.52 ^a^
VL-17A	7.68 ± 0.22 ^a^	27.15 ± 0.57 ^a^	6.48 ± 0.56 ^a^	27.65 ± 1.19 ^a^

^a^ Data is presented as the mean percentage of cells ± the SE. *n* = 4.
